# Effectiveness of a telehealth physiotherapist-delivered intensive dietary weight loss program combined with exercise in people with knee osteoarthritis and overweight or obesity: study protocol for the POWER randomized controlled trial

**DOI:** 10.1186/s12891-022-05685-z

**Published:** 2022-07-30

**Authors:** Kim L. Bennell, Sarah E. Jones, Rana S. Hinman, Fiona McManus, Karen E. Lamb, Jonathan G. Quicke, Priya Sumithran, Jodie Prendergast, Elena S. George, Melanie A. Holden, Nadine E. Foster, Kim Allison

**Affiliations:** 1 Centre for Health, Exercise and Sports Medicine, Department of Physiotherapy, The University of Melbourne, Melbourne, Australia; 2 Centre for Epidemiology and Biostatistics, Melbourne School of Population and Global Health, The University of Melbourne, Melbourne, Australia; 3 Methods and Implementation Support for Clinical Health research Hub, Faculty of Medicine, Dentistry and Health Sciences, The University of Melbourne, Melbourne, Australia; 4 School of Medicine, Keele University, Keele, UK; 5 Chartered Society of Physiotherapy, Chancery Exchange, London, UK; 6 Department of Medicine, The University of Melbourne, Melbourne, Australia; 7 Medibank Private, Melbourne, Australia; 8 Institute for Physical Activity and Nutrition, School of Exercise and Nutrition Sciences, Deakin University, Melbourne, Australia; 9 STARS Education and Research Alliance, Surgical Treatment and Rehabilitation Service (STARS), The University of Queensland and Metro North Health, Brisbane, Australia

**Keywords:** Osteoarthritis, OA, Knee, Telehealth, Overweight, Obesity, Weight management, Physiotherapy, Ketogenic diet, Exercise, Physical activity, Clinical trial, RCT

## Abstract

**Background:**

Obesity is associated with knee osteoarthritis (OA). Weight loss, alongside exercise, is a recommended treatment for individuals with knee OA and overweight/obesity. However, many patients cannot access weight loss specialists such as dietitians. Innovative care models expanding roles of other clinicians may increase access to weight loss support for people with knee OA. Physiotherapists may be well placed to deliver such support. This two-group parallel, superiority randomized controlled trial aims to compare a physiotherapist-delivered diet and exercise program to an exercise program alone, over 6 months. The primary hypothesis is that the physiotherapist-delivered diet plus exercise program will lead to greater weight loss than the exercise program.

**Methods:**

88 participants with painful knee OA and body mass index (BMI) > 27 kg/m^2^ will be recruited from the community. Following baseline assessment, participants will be randomised to either exercise alone or diet plus exercise groups. Participants in the exercise group will have 6 consultations (20–30 min) via videoconference with a physiotherapist over 6 months for a strengthening exercise program, physical activity plan and educational/exercise resources. Participants in the diet plus exercise group will have 6 consultations (50–75 min) via videoconference with a physiotherapist prescribing a ketogenic very low-calorie diet with meal replacements and educational resources to support weight loss and healthy eating, plus the intervention of the exercise only group. Outcomes are measured at baseline and 6 months. The primary outcome is percentage change in body weight measured by a blinded assessor. Secondary outcomes include self-reported knee pain, physical function, global change in knee problems, quality of life, physical activity levels, and internalised weight stigma, as well as BMI, waist circumference, waist-to-hip ratio, physical performance measures and quadriceps strength, measured by a blinded assessor. Additional measures include adherence, adverse events, fidelity and process measures.

**Discussion:**

This trial will determine whether a physiotherapist-delivered diet plus exercise program is more effective for weight loss than an exercise only program. Findings will inform the development and implementation of innovative health service models addressing weight management and exercise for patients with knee OA and overweight/obesity.

**Trial registration:**

NIH US National Library of Medicine, Clinicaltrials.gov NCT04733053 (Feb 1 2021).

## Background

The prevalence and burden of obesity is escalating worldwide [1]. Obesity is linked to many health conditions [2], including osteoarthritis (OA), a leading cause of chronic pain and disability [3] and affecting one in five Australians over the age of 45 years [4]. Overweight and obesity is a significant risk factor for the development [5] and progression [6] of knee OA and is associated with an increased likelihood of knee joint replacement surgery [7]. Evidence shows that losing at least 5–10% of body weight provides clinically important improvements in knee pain and function [8–10] and reduces the likelihood of knee joint replacement [11, 12], with greater benefits when dietary weight loss is combined with exercise [8, 13]. Thus, clinical guidelines for knee OA consistently recommend weight loss for those who have overweight or obesity, together with exercise, as a core treatment [14, 15]. However, many patients do not receive any weight loss support from healthcare practitioners [16–18] and there are currently insufficient numbers of, and referrals to, practitioners with specialist weight loss skills, such as dietitians [16]. Innovative models of care that expand practice roles of other healthcare practitioners [19] may help increase access to effective weight loss support for people with knee OA and address this unmet clinical need.

Physiotherapists are primary care providers of therapeutic exercise for people with knee OA and are thus well placed to play a greater role in weight loss support [20]. From a workforce capacity perspective, there are many more practicing physiotherapists than dietitians. For example in Australia, physiotherapists outnumber dietitians in the community by over 4:1 [21, 22]. As exercise and rehabilitation specialists who already manage people with knee OA and often develop strong patient rapport [23, 24], there is opportunity for physiotherapists to integrate and support exercise and dietary weight loss together for synergistic benefit [8, 13]. This also obviates the need for patients to consult multiple practitioners for these treatments which may not be possible due to availability, proximity and cost and which can be time-consuming and less convenient. Physiotherapists also have the advantage of having relatively longer consultation times and often more frequent patient follow-up than other healthcare practitioners, such as family doctors, which may allow greater opportunity for weight loss [20]. Furthermore, the physiotherapy profession has previously demonstrated flexibility in adapting to changes in other health care models beyond their traditional scope of practice, such as by providing psychologically-informed care for complex pain [25].

To play a greater role in weight management over and above simply providing advice about the role of weight in OA, physiotherapists require upskilling as many currently lack the requisite knowledge, skills and confidence to prescribe and support a dietary weight loss intervention [26, 27]. We have shown that a customized self-directed, relatively short (< 10 hours) e-learning program for physiotherapists can substantially increase their confidence in both their knowledge and clinical skills in weight management for patients with knee OA [28]. However, further research is needed to determine whether physiotherapists can use this new knowledge to effectively and safely deliver a dietary weight loss intervention, in addition to other core OA treatments of education and exercise that are within traditional scope of physiotherapy practice.

While knee OA clinical guidelines recommend weight loss [14, 15], they do not specify how best to do this or which dietary approach to use. There is evidence to show that very low calorie diets (VLCDs) involving meal replacements are more effective for inducing significant weight loss and improving physical function than diets that involve lifestyle counselling alone in people with knee OA [29]. Ketogenic VLCDs, whereby carbohydrate intake is additionally restricted (< 50-60 g per day), have also been demonstrated to be an effective and safe means of achieving rapid weight loss in the adult population with overweight and obesity [30] and lead to greater weight loss than low-fat diets in the short-term [31]. An advantage of a ketogenic diet is possible reduction in hunger [32, 33], a feature that may assist with adherence to caloric restriction. The predicted rapid weight loss of 1.5–2.5 kg per week [31] means that those adhering to a ketogenic VLCD frequently achieve a 10–15% weight loss target weight within 12 weeks, an amount likely to have symptomatic benefits for people with knee OA [13].

In a recent randomized controlled trial (RCT), we tested the effectiveness of a telehealth program comprising a ketogenic VLCD delivered by dietitians and exercise delivered by physiotherapists for people with knee OA. The trial showed that this combined program led to greater improvements in pain and function, as well as greater weight loss, when compared to an exercise-only program and to online education control [34]. In our qualitative research, participants described positive experiences with the ketogenic VLCD program, valuing its simplicity and effectiveness [35]. Support from dietitians and a comprehensive suite of educational and behaviour change resources, incorporated with an exercise program, were considered crucial for weight loss success. The telehealth delivery mode was valued for its convenience [35, 36], particularly time efficiency and access. The protocolized and nutritionally complete manner of the ketogenic VLCD using meal replacements means that healthcare practitioners without formal nutrition qualifications, such as physiotherapists, may be capable of effectively and safely supporting such a VLCD for some people with knee OA.

This trial aims to evaluate the effectiveness of a 6-month telehealth physiotherapist-delivered ketogenic VLCD added to exercise compared with telehealth physiotherapist-delivered exercise alone in people with knee OA who have overweight/obesity. The primary hypothesis is that the group offered the diet plus exercise program will lose more weight than the group offered an exercise only program at 6 months. The trial will also evaluate effects on secondary outcomes including body mass index, weight circumference, waist-to-hip ratio, knee pain, physical function, global change in knee problem, quality of life, physical activity levels, internalized weight stigma, physical performance and quadriceps muscle strength. Additional measures will assess adherence, adverse events, intervention fidelity and a range of process outcomes.

## Methods

### Study design

The POWER trial is a two-group parallel, superiority RCT based in Melbourne, Australia. The University of Melbourne Human Research Ethics Committee approved the study (HREC 1955042 ref. 2022–13,143–27,079-5) and the University of Melbourne (Victoria, Australia) is the trial sponsor. The study sponsor will not have any role in study design; collection, management, analysis, and interpretation of data; writing of the final report or the decision to submit the report for publication. The trial was prospectively registered with Clinicaltrials.gov (NCT04733053). This protocol was reported according to the Standard Protocol Items: Recommendations for Interventional Trials statement [37] and trial findings will be reported based on the Consolidated Standards of Reporting Trials statement (CONSORT non-pharmacological treatment interventions) [37] and Template for Interventional Description and Replication (TIDier) [38] guidelines. A trial Data Safety and Monitoring Committee was established consisting of a doctor with expertise in weight loss, a dietitian, a biostatistician and a physiotherapist in clinical practice, none of whom are involved in the trial, have any conflicts of interest or will benefit from the results. The committee will receive a study report every 3 months but will not meet unless warranted. The responsibility of the committee will be to provide recommendations to the chief investigator about continuing, modifying or stopping the trial. There is no planned interim analysis or stopping guidelines.

### Participants

We will recruit 88 participants with chronic knee pain, consistent with a clinical diagnosis of knee OA, from the community in Victoria (Australia) via advertisements, print/radio/social media and our volunteer database. Participants will be included if they:

i) meet the National Institute for Health and Care Excellence [39] clinical criteria for OA;
  - age ≥ 45 years;
  - report activity-related knee joint pain;
  - report no knee morning stiffness or morning knee stiffness lasting ≤30 mins
ii) report a history of knee pain ≥3mths;
iii) report knee pain on most days of the past month;
iv) report a minimum knee pain score during walking over the past week of 4 on an 11-point numeric rating scale (NRS);
v) have a body mass index (BMI) > 27 kg/m^2^ [40];
vi) are willing to check their blood pressure (by self or pharmacist/general practitioner) if they are using hypertensive medication and feel light-headed or dizzy during the trial;
vii) are able to give informed consent and to participate fully in the interventions and assessment procedures.

Participants are excluded if they:

i) are aged over 80 years;
ii) weigh > 150 kgs (due to the complexity of additional nutritional requirements for individuals above this weight);
iii) are unable to speak English;
iv) are on a waiting list for/planning knee/hip surgery or bariatric surgery in next 6 months;
v) have had previous arthroplasty on the affected knee;
vi) report recent knee surgery on affected knee (past 6 months);
vii) report inflammatory arthritis (e.g. rheumatoid arthritis);
viii) have had weight loss of > 2 kg over the previous 3 months;
ix) are already actively trying to lose weight by any of the following:
  - using formulated meal replacements;
  - being a member of a slimming club (such as Weight Watchers);
  - receiving support from another healthcare practitioner;
  - using prescribed weight loss drugs;
  - using structured meal programs (such as ‘Lite n’ Easy’);
x) are unwilling to continue current dietary patterns if randomized to the exercise group;
xi) are unable to undertake a ketogenic VLCD without close medical supervision because of the following self-reported conditions:
  i) Type 1 diabetes;
  ii) Type 2 diabetes requiring medication apart from metformin;
  iii) warfarin use;
  iv) stroke or cardiac event in previous 6 months;
  v) unstable cardiovascular condition;
  vi) fluid intake restriction;
  vii) renal (kidney) problems (unless clearance is obtained from general practitioner, including confirmation that the estimated glomerular filtration rate is > 30 mL/min/1.73m^2^);
xii) have any neurological condition affecting lower limbs;
xiii) have vegan dietary requirements (due to complexity of delivering a nutritionally complete diet within the ketogenic VLCD).

### Procedures overview

**Fig. 1:**
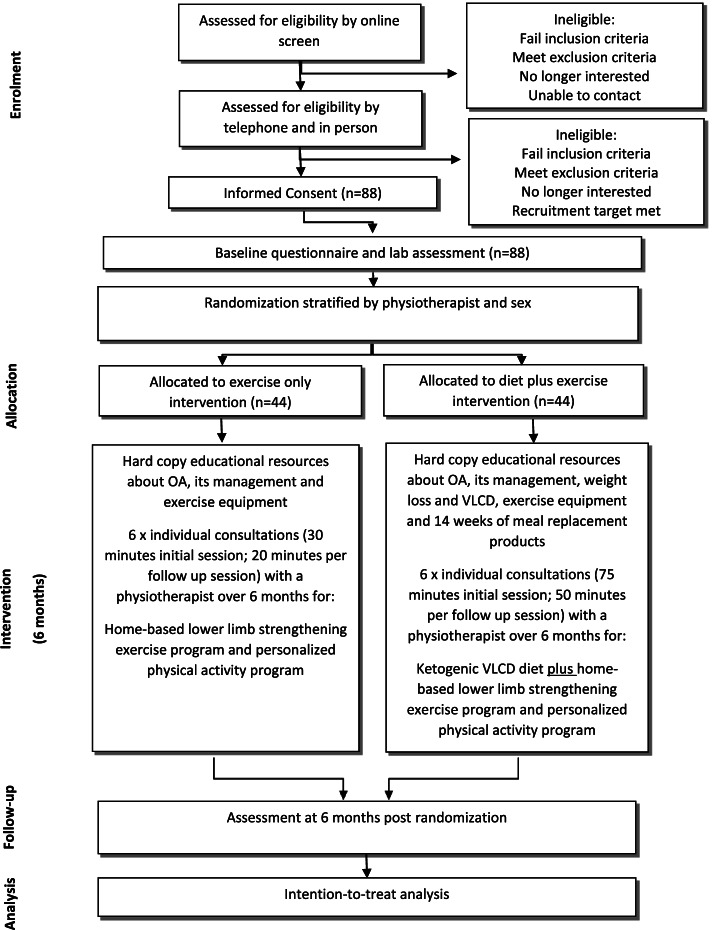
Flow diagram summarizing flow of participants through study phases

The trial phases are summarized in Fig. 1. Volunteers will undergo screening via an online form then over the telephone by a researcher. Additional clearance to participate will be sought from a general practitioner for anyone who indicates that they have renal dysfunction. A detailed verbal description of the trial including the purpose, aims, possible side effects, content of the interventions and trial commitments will be provided by the researcher during the telephone screening process. Volunteers who are eligible following telephone screening will be sent the Plain Language Statement and Consent Form via email or post by the trial coordinator and will be encouraged to phone the researchers if they have any questions or concerns regarding their contents. After obtaining their written informed consent, either online (REDCap™) or on paper, participants will undergo assessment at the University, including confirmation of weight and BMI eligibility, and complete baseline questionnaires either online (REDCap™) or on paper. A secure data collection platform (Qualtrics or REDCap™) accessible only by password to the researchers will store screening information, trial consent forms and all outcome data. For participants with bilaterally eligible knees, the most symptomatic knee will be deemed the trial knee with respect to outcome measurement. If both knees are equally symptomatic, then the right knee will be chosen. Efforts will be taken to minimize loss of data, including collection of self-reported primary outcome data over the telephone in cases where participants are unable to attend the in person follow up assessment at 6-months.

### Randomization, blinding and allocation concealment

Eligible participants will be randomised to receive either i) exercise alone or ii) diet plus exercise. The randomization schedule will be computer-generated and prepared by an independent biostatistician. The first list will randomly allocate participants to a physiotherapist (no strata). The second list will randomly allocate participants to an intervention group using variable permuted block sizes and a randomization ratio of 1:1, stratified by physiotherapist and participant sex (due to sex differences in weight gain, weight loss, and attitudes to weight loss [41, 42]). If a physiotherapist is unavailable (e.g. sick, on holiday) participants will be re-randomized to another available physiotherapist using a third list with variable permuted block sizes and stratified by group allocation and sex. The randomization schedule will be stored on a password-protected website (REDCap™) at the University of Melbourne and maintained by a researcher not involved in either participant recruitment or administration of primary/secondary outcome measures. Group allocation is revealed by this same researcher after completion of baseline assessment.

The components of each intervention will be disclosed to participants prior to enrolment as we wish to test the interventions under real world conditions whereby consumers are fully informed about their treatment components before choosing whether they wish to participate. The research staff member collecting non self-reported outcome measures (including the primary outcome) at 6 months will be blinded to group allocation and will be a different person from the one collecting baseline data. This is necessary given that obvious weight loss has the potential to unblind the assessor if baseline and 6 month measures were to be collected by the same person. It is not possible to blind physiotherapists as they are providing interventions to participants in both trial arms to ensure physiotherapist-related factors such as personality or clinical experience are similar between groups and cannot confound results. The statistical analysis plan will be written and published while the biostatisticians are blinded.

### Physiotherapists and training

We will use our network of physiotherapists and advertisements in the Australian Physiotherapy Association fortnightly e-news bulletin to recruit six practicing musculoskeletal physiotherapists working in private practice in Melbourne, Australia. These physiotherapists will deliver care to participants randomized to both trial groups. Eligibility criteria for the physiotherapists to deliver the interventions in this trial are:

- Current registration to practice as a physiotherapist with the Australian Health Practitioner Regulation Agency;
- A receptionist at their clinical practice to facilitate participant bookings and communication with research staff;
- An Australian Business Number;
- Willing to undertake trial training requirements;
- Willing and available to deliver interventions until the anticipated completion of the trial.

Mandatory comprehensive training (estimated total time commitment of 20 hours) will be undertaken by physiotherapists prior to them being allocated a trial participant. The training components include:

- Bespoke self-directed e-learning modules (on the University of Melbourne Learning Management System) covering: best practice OA management; strengthening exercises and physical activity prescription including the structured trial protocol and resources for use in the exercise component; the link between overweight/obesity and OA; having a weight loss conversation; weight stigma in physiotherapy; lifestyle and dietary interventions for weight loss; background to the ketogenic VLCD and the structured trial diet intervention protocol including safety issues; behaviour change support techniques and motivational interviewing techniques to promote adherence as well as resources for use in the diet component; telehealth (including delivery of care via Zoom video-conferencing), and study procedures including consultation structure. Physiotherapists will be told it will take approximately 10–12 hours to work through all e-learning modules, which they are encouraged to complete at their own pace over 6 weeks. The general e-learning modules (not specific to the study procedures) have since been adapted, and released for widespread use by clinicians outside of the trial and access is available to users globally:

(www.futurelearn.com/courses/eduweight).

- Six videoconference consultations to deliver sessions 1, 2 and 4 (one week apart) of the diet plus exercise program to one ‘mock’ patient (one of the research team) and one ‘practice’ patient with knee OA (recruited from our network). The sessions will be audiotaped. The researcher will provide feedback to the physiotherapist on competency in telehealth delivery and adherence to the protocol using a checklist of 60 items. The researcher will also discuss the consultations with the practice patient and provide feedback to the physiotherapist from the patient’s perspective.
- Teleconference(s) with the trial coordinator and a trial dietitian to answer any questions and clarify any study procedures with an estimated time commitment of one hour.

A hard copy of a “Physiotherapist Manual” detailing intervention protocols and study procedures, along with a hard copy of each of the participant resources, will be provided to each physiotherapist for their own reference and to facilitate study consultations.

### Interventions

Participants in both groups will consult a physiotherapist using the videoconferencing facility of Zoom (Zoom Video Communications, Inc., USA) for six individual sessions over 6 months. The consultations are recommended to occur in weeks 1, 3, 7, 11, 15 and 19, but the precise timing can be decided between each participant and their physiotherapist. Research staff will book the first two appointments on behalf of participants. Thereafter, physiotherapists will negotiate with participants to book follow-up consultations. The same physiotherapist will ideally undertake all consultations with any given participant. Before their first consultation with the physiotherapist, participants will complete a pre-consultation survey online asking about their main problems and goals, a brief history of their knee symptoms, weight and diet patterns (for the diet plus exercise group), and other health problems. All participants will also be provided with hard copy OA educational resources, activity booklets and log books, and resistance bands to facilitate their engagement with their program. Resources provided to both groups are detailed in Table 1.

**Table 1:** Summary of resources provided to participants in the Exercise group and Diet plus exercise group

Resource	Description	Exercise group	Diet plus exercise group
Exercise resistance bands	4 exercise resistance bands (red, green, blue, black) to increase resistance for strengthening exercise	✓	✓
Formulated meal replacements	14 weeks of Optifast (or Optislim if vegetarian or Optifast unavailable) meal replacement products for ketogenic VLCD		✓
Plastic portion plate	Visual aid to manage portion sizes		✓
**Booklets**			
Preparing for your consultations	Information about consultations, instructions about how to download and use Zoom video conferencing	✓	✓
Exercise booklet	Step-by-step photographs and instructions for strengthening exercises for quadriceps, hip abductors, hamstrings, gluteal and calf muscles and balance exercises	✓	✓
Knee plan and log book	Workbook containing templates to detail agreed exercise plan and to track exercise sessions completed each week	✓	✓
OA information	Evidence-based information covering understanding OA, management options, misconceptions, pain coping skills and sleep	✓	✓
Weight management “how to” guide	Information on how to undertake the VLCD and how to transition from VLCD to normal healthy eating		✓
Weight management activities	Workbook containing behavioral support activities including finding a support person, tracking weight, identifying eating triggers, changing thought patterns, monitoring hunger levels and overcoming barriers		✓
Recipe book	A selection of ~ 75 recipes suitable for low glycaemic index carbohydrate meals or VLCD		✓
Food list pocket guide	Laminated list of low carbohydrate whole foods appropriate to eat during the ketogenic VLCD		✓

#### a) Exercise program

Physiotherapist consultations for participants in this group will last 30 minutes initially and 20 minutes thereafter. Physiotherapists will prescribe a home strengthening exercise program to be performed by the participant at home three times per week. The exercise program was used previously and shown to be effective [34, 43, 44]. The program will include 5–6 strengthening exercises from a pre-determined list including two quadriceps exercises, one each for the hip abductors, hamstrings and calf muscles, and any other exercise as appropriate from the list (Table 2) [43–46]. Participants will be asked to exercise at a moderate intensity (self-perceived effort of ≥5 out of 10 (hard) on a modified Borg Rating of Perceived Exertion scale [47]). So that they can provide real-time demonstration of exercises to participants during the videoconferencing consultations, using the share-screen feature of Zoom, physiotherapists are given access to a bespoke website containing a video library of exercises contained within the “Exercise Booklet”. The physiotherapist will also prescribe and support a personalized and progressive physical activity plan in collaboration with the participant. The physiotherapist will help the participant to identify barriers to exercise and possible solutions, advise on how to self-monitor and manage any pain flare-ups, and review, modify and progress the program as appropriate.

**Table 2:** Strengthening exercise protocol, with instructions and progressions where applicable. Reprinted from: Hinman RS, Kimp AJ, Campbell PK, Russell T, Foster NE, Kasza J, Harris A, Bennell KL: Technology versus tradition: a non-inferiority trial comparing video to face-to-face consultations with a physiotherapist for people with knee osteoarthritis. Protocol for the PEAK randomised controlled trial. BMC Musculoskelet Disord 2020, 21(1):522 [45]

Home exercise protocol			
**Maximum of 6 exercises** (with progression as appropriate) performed three times a week - 2 knee extensor strengthening exercises - 1 hip abductor strengthening exercise - 1 hamstring strengthening exercise - 1 calf strengthening exercise - 1 other exercise as appropriate from the list (optional) **Dosage** Determined on an individual basis by the physiotherapist			
**1. Quads strengthening (aim to include two exercises)**			
**Knee extension**	Non weight-bearing	Q1. Seated knee extension (with resistance) with 5 s hold	**Progression:** Increase with theraband resistance – red through to black
	Non weight-bearing	Q2. Inner range quads over roll (with resistance) with 5 s hold	**Instruction:** Put a rolled up towel under your arthritis knee. Keep the knee cap and toes pointing toward the roof. Keeping the back of the knee in contact with the towel, push the back of your knee down into the towel and straighten your arthritis leg
**Sit-to-stand**	Weight-bearing	Q3. Sit to stand (without using hands)	**Progression:** lower chair height, hover above the seat without touching down (3 s hold), add resistance band around knees and push outwards keeping knees over toes
	Weight-bearing	Q4. Sit to stand with more weight on involved knee	**Instruction:** Take more weight by either a) placing uninvolved further forward b) shift both feet sideways so study leg is midline
**Steps**	Weight-bearing	Q5. Step-ups	**Progression:** Increase step height
	Weight-bearing	Q6. Forward touchdowns from a step	**Progression:** Increase step height, don’t touch floor
	Weight-bearing	Q7. Step-ups with weight	**Instruction:** Hold 2 kg of weight either a) against chest, b) in each hand, c) in one hand while holding for balance, or d) in backpack **Progression:** Increase step height, increase weight
	Weight-bearing	Q8. Forward touchdowns from a step with weight	**Instruction:** Hold 2 kg of weight either a) against chest, b) in each hand, c) in one hand while holding for balance, or d) in backpack **Progression:** Increase step height, increase weight
**Wall squats**	Weight-bearing	Q9. Partial wall squats for 5 s hold	**Progression:** more weight on study side
	Weight-bearing	Q10. Split leg wall squats for 5 s hold	**Instruction:** Step feet away from wall (about 30 cm) and move non-involved leg a further 15 cm away from the wall.
**Controlled squats**	Weight-bearing	Q11. Controlled partial squat with 5 s hold	
**Leg Sliding**	Weight-bearing	Q12. Forward and backward sliding of non-study leg	**Instruction:** Keep weight on study leg. Concentrate on keeping knee positioned over the foot
	Weight-bearing	Q13. Forward and backward sliding of non-study leg with resistance band pulling study leg laterally	**Instruction:** Place loop of elastic band around involved knee and leg of a table to provide a pull outwards on knee that you must resist by keeping knee in line with foot. **Progression:** Increase with theraband resistance – red through to black
	Weight-bearing	Q16. Sideways sliding of uninvolved leg	**Instruction:** Keep weight on study leg. Concentrate on keeping knee positioned over the foot
	Weight-bearing	Q17. Sideways sliding of uninvolved leg with resistance band pulling study leg laterally	**Instruction:** Place loop of elastic band around study leg and leg of a table to provide a pull outwards on knee that you must resist by keeping knee in line with foot. **Progression:** Increase with theraband resistance – red through to black
**Step to single leg balance**	Weight-bearing	Q14. Step with study leg to about 30° knee flexion for single leg balance	**Instruction:** Take a step forwards with study leg, keeping knee bent to about 30° knee flexion. Allow uninvolved leg to lift off and practice balancing as long as you can
	Weight-bearing	Q15. Step with study leg to about 30° knee flexion for single leg balance with arm movements	**Instruction:** Take a step forwards with involved knee, keeping knee bent to about 30° knee flexion. Allow uninvolved leg to lift off and practice balancing as long as you can, while raising arms out to side and above head in an arc.
**2. Hip abductor strengthening (1 exercise)**			
**Standing hip abduction**	Weight-bearing	HA1. Side leg raises in standing with 5 s hold	**Progression**: Increase theraband resistance – red through to black, add another 5 s halfway
**Side stepping**	Weight-bearing	HA2. Crab walk with resistance band	**Progression:** Increase theraband resistance – red through to black
**Standing hip abduction**	Weight bearing	HA3. Wall push standing on study leg for 20 s	**Progression:** Hold weight in hand, increase the hold time
	Weight bearing	HA4. Wall push standing on study leg positioned up to 45° knee flexion	
**3. Hamstring strengthening (1 exercise)**			
**Bridging**	Weight-bearing	HG1. Bridge with 5 s hold	
	Weight-bearing	HG2. Split leg bridge with 5 s hold	**Instruction:** Place feet hip-width apart, then move study leg slightly closer toward your bottom and slightly in towards centre
	Weight-bearing	HG3. Single-leg bridge on study leg with 5 s hold	**Version A:** Lift bottom. Keeping hips level, lift uninvolved leg off the floor/bed. Hold for 5 s. Slowly lower uninvolved leg back to the floor. Then slowly lower bottom back to floor. **Version B:** Lift uninvolved knee off floor/bed. Lift bottom and take all weight through study leg. Hold for 5 s. Slowly lower your bottom back to floor/bed.
**Standing knee flexion**	Non weight-bearing	HG4. Hamstring curls -Standing over bench knee flexion with 5 s hold	
	Non weight-bearing	HG5. Hamstring curls -Standing over bench knee flexion with 5 s hold against resistance band	**Instruction:** Place one end of an elastic band securely around ankle of study knee. Place the other end of elastic around your opposite foot so you are standing on it.
**Seated knee flexion**	Non weight-bearing	HG6. Seated knee flexion	**Instruction:** Place one end of an elastic band securely around a stable object (e.g. a heavy table leg). Loop the other end around the ankle of your study leg. Keeping your opposite foot on the floor, pull against the elastic band and bend your knee more. **Progression:** Increase with theraband resistance – red through to black
**Standing hip extension**	Non weight-bearing	HG7. Hip extension with knee bent (90°) - standing over bench with 5 s hold	
	Non weight-bearing	HG8. Hip extension with knee straight - standing over bench with 5 s hold	
	Non weight-bearing	HG9. Hip extension with knee straight with resistance band - standing over bench with 5 s hold	**Instruction:** Place one end of an elastic band securely around ankle of study knee. Place the other end of elastic around your opposite foot so you are standing on it.
**4. Calf strengthening (1 exercise)**			
**Standing plantar-flexion**	Weight-bearing	C1. Double heel raises with 5 s hold	
	Weight-bearing	C2. Single heel raises with 5 s hold	
	Weight-bearing	C3. Double heel raises with 5 s hold over edge of step	
	Weight-bearing	C4. Double heel raises with 5 s hold over edge of step	
**5. Balance exercises**			
**Tandem stance**	Weight- bearing	B1. Maintain balance in tandem stance	**Progression:** remove hand support, slowly raise arms in the air, eyes closed
**Natural stance**	Weight- bearing	B2. Maintain balance whilst tapping opposite foot forwards & backwards	
**Single leg stance**	Weight- bearing	B3. Maintain balance in single leg stance	**Progression:** Increase hold time, slowly raise arms up and down, eyes closed

#### b) Diet plus exercise program

Physiotherapist consultations for participants in this group will last 75 minutes initially (30 minutes for the exercise component and 45 minutes for the diet component) and 50 minutes thereafter (20 minutes for the exercise component and 30 minutes for the diet component). The exercise components for this group are the same as those described above for the exercise only program.

The physiotherapist will support the participant to lose weight (aiming for ≥10% body weight loss) via a ketogenic VLCD, with additional dietary/behavioural educational resources provided in hardcopy [34] (Table 1). Ahead of their initial consultation, participants will be sent approximately 4–5 weeks of meal replacement products from the Optifast product range (Nestlé Health Science, Rhodes, Australia). In cases where Optifast products are out of stock, or the participant cannot tolerate or does not wish to consume products containing fish oil, Optislim meal replacements are provided as an alternative (Optipharm, Australia). Participants will be asked to register their product and flavour preferences for each of their orders. Meal replacements will continue to be supplied at no cost to the participant for a maximum of 14 weeks.

Participants will collaboratively develop a weight management plan and a weight loss goal with their physiotherapist during their initial consultation. Physiotherapists will ask participants to substitute two of their normal meals each day with meal replacement products, with the participants able to choose which meal they substitute with which product. For their third meal of the day, participants are asked to consume a low carbohydrate, low fat meal, consisting of high-quality protein (e.g. meat/fish/eggs/tofu), non-starchy vegetables, and (for participants who have not had their gall bladder removed) the equivalent of a tablespoon of unsaturated fat (e.g. olive oil/ nuts/avocado). Importantly total caloric intake should be approximately 800 kcal (3280 kJ) per day and carbohydrate intake should not exceed 50–60 g per day. Modifications of the diet will be permitted as necessary in instances where participants are unwilling to stick to the diet as prescribed. In such cases, caloric restriction and low carbohydrate intake will continue to be advised. Participants will be encouraged to track their weight on a weekly basis and are provided space to do so in their weight management activities booklet.

During the subsequent five consultations, participants will self-report their progress, and any challenges or difficulties they have experienced with their weight management plan. Physiotherapists will use motivational interviewing techniques to help encourage self-efficacy and motivation, and collaboratively develop ways to help the participant overcome their barriers. Physiotherapists will encourage participants to engage with activities in the booklets including setting realistic goals; keeping a food diary; identifying a suitable support person; identifying eating triggers; developing strategies to deal with challenging situations and monitoring and mindfulness of hunger levels, as well as discussing education topics such as healthy foods and portion sizes.

Physiotherapists will support participants in deciding when to transition off the ketogenic VLCD, first raising the discussion when they have achieved 10% weight loss, or once they have been on the ketogenic diet for 12 weeks, whichever comes first. During the two-week transition phase, participants will be asked to consume only one meal replacement per day and to gradually reintroduce low glycaemic index carbohydrates to their other meals. After the two week transition phase, participants will enter the weight maintenance phase in which they will be asked to eat a healthy whole food diet, focusing on meals which are high in protein, low in fat and with low glycaemic index carbohydrates, as recommended by the Commonwealth Scientific and Industrial Research Organisation Total Wellbeing diet [48]. Participants will receive verbal education and support from the physiotherapist during their remaining physiotherapy consultations to maintain their weight loss. The hard copy resources which they keep once their involvement in the study is complete also contains this information. Participants will be advised by the physiotherapist to continue monitoring their weight on a weekly basis, and if they gain 2 kg or more, to return to the ketogenic VLCD for 1–2 weeks.

Meal replacements will be provided to participants at no cost for the first 14 weeks of the study.

After this time, meal replacements will be self-sourced and self-funded if participants opt to re-engage with the ketogenic VLCD. Participants will be offered strategies and resources during the program, which will assist their long-term weight maintenance after their consultations with the physiotherapist are completed, including regular weight monitoring and dealing with setbacks [34].

### Outcome measures

Body weight will be measured at baseline and 6 months on the same set of calibrated digital high capacity scales (Seca 813). Weight will be reported in kgs to two decimal places. Change in body weight, expressed as a percentage, is the primary outcome.

There are a number of secondary outcomes. These are measured at baseline and 6 months, unless otherwise stated, and include:

i) BMI in kg/m^2^;
ii) Waist circumference at mid-abdomen level at its smallest circumference in cm;
iii) Waist-to-hip ratio by dividing the waist circumference by the hip circumference at its widest part;
iv) Self-reported average knee pain on walking in the last week measured using an 11-point NRS with terminal indicators of ‘no pain’ = ‘0’ and ‘worst pain possible’=‘10’ [49];
v) Intermittent and constant osteoarthritis pain measure (iCOAP) [50] with 11 items using a 4-point Likert scale and subscales for constant pain (scored 0 to 20) and intermittent pain (scored 0 to 24). Higher scores indicate higher levels of pain;
vi) Physical function subscale of the Western Ontario and McMaster Universities Osteoarthritis Index (WOMAC) [51] scored over 17 items, using a 4-point Likert scale with a total score between 0 and 68. Higher scores indicate more severe dysfunction;
vii) Perceived global change in knee problems scored on a 7-point Likert scale from ‘much worse’ to ‘much better’ [52] at the 6-month time point only. Participants who indicate that they are “moderately better” or “much better” will be categorized as ‘improved’. All other respondents will be categorized as ‘not improved’;
viii) Health-related quality of life using the Assessment of Quality of Life (AQoL) (version AQoL-6D) [53], a 20-item instrument with scores ranging between − 0.04 to 1.0 and higher scores indicating higher quality of life;
ix) Physical activity levels evaluated using the physical activity scale for the elderly (PASE) [54] with scores from 0 to 400 with higher scores indicating greater levels of physical activity;
x) Weight Self-Stigma Questionnaire (WSSQ) to evaluate internalized weight stigma [55] reported via two subscales (self-devaluation and fear of enacted stigma) and a combined total of 0–60 with higher scores indicating greater internalized weight stigma;
xi) Physical performance measures recommended by the Osteoarthritis Research Society International [56] and assessed by a researcher blinded to group allocation;

- 30s chair sit-to-stand test: The number of complete chair stands achieved in 30s will be reported [56]. Higher scores indicate better physical function.
- 40 m fast-paced walk test: The total time taken to walk 40 m quickly but safely will be recorded, and reported in speed (m/s) [56]. Faster walking speeds indicate better physical function.
- 6-step stair climb test: The total time taken to ascend and descend a flight of six stairs will be recorded [57]. Use of the handrail is optional. Shorter completion times indicate better physical function.

xii) Maximum voluntary isometric strength of the knee extensors measured on an isokinetic dynamometer (HUMAC, CSI, Boston) with the knee held at 60 degrees flexion. The maximum torque reached over 3 repetitions of 5 seconds, normalized to body mass, will be recorded in Nm/kg.

### Adherence measures

A number of adherence measures will be collected.

i) Number of physiotherapist consultations attended for each participant (possible range 0–6);
ii) Self-reported number of home exercise sessions in the last two weeks (options of 0, 1, 2, 3, 4, 5 and 6+) at 6 months and converted to a percentage out of the 6 prescribed sessions.
iii) Self-rated adherence to the home strengthening program scored on an 11-point NRS in response to the question “I have been doing my exercise sessions the number of times I was asked to by my POWER physiotherapist (e.g. three times per week)” over the previous 6 months where 0 = strongly disagree and 10 = strongly agree;
iv) Self-rated adherence with the physical activity plan scored on an 11-point NRS in response to the question “I followed the physical activity plan that my POWER trial physiotherapist helped me to develop” over the previous 6 months where 0 = strongly disagree and 10 = strongly agree;
v) Self-rated adherence to the diet program (diet plus exercise group only) scored on an 11-point NRS in response to the question “I followed the diet plan as it was outlined by my POWER trial physiotherapist” over the previous 6 months where 0 = strongly disagree and 10 = strongly agree.

### Intervention fidelity

Physiotherapists will complete bespoke semi-structured electronic treatment notes for each consultation and submit these for review by research staff to ensure adherence to trial intervention protocols. Fidelity will be reported as: i) duration in minutes of each consultation recorded by the physiotherapist; ii) number and proportion of consultations where the physiotherapist has undertaken specific required elements of the intervention protocol (such as set weight loss goal, assessed progress, discussed different educational topics, prescribed exercises, recorded weight).

### Process and other measures

These include:

i) Physiotherapists’ contact with trial investigators for advice or support relating to the weight loss program during delivery of the intervention will be recorded by the researchers. Number of physiotherapists who contacted the trial coordinator, number of contacts and reasons for contact will be reported;
ii) Participant’s satisfaction with their allocated intervention assessed using a seven-point global rating scale with options ranging from ‘extremely dissatisfied’, to ‘extremely satisfied’. Participants who indicate that they are moderately or extremely satisfied will be categorized as satisfied;
iii) Therapeutic alliance assessed using the Working Alliance Inventory Short Form [58]. This will be completed by participants at 6-months while physiotherapists will complete this following the last consultation with the participant. Overall scores for each range from 12 to 84, with higher scores indicating a stronger therapeutic alliance;
iv) The attitude of participants towards different clinicians delivering weight loss interventions assessed at baseline and 6 months by response to the statement “I am confident that a [general practitioner / dietician / physiotherapist] could deliver a dietary weight loss program” using a 5-point Likert Scale for each clinician, with terminal indicators of 1 = strongly disagree, to 5 = strongly agree;
v) Participants allocated to the diet plus exercise group will be asked to report the total number of weeks they used meal replacements for over the 6 months.
vi) Participants allocated to the diet plus exercise group will be asked to respond to 8 custom statements exploring their perception of the knowledge and skills of their physiotherapist and their confidence in, and comfort working with, the physiotherapist for delivery and support of the dietary weight loss program, at the 6 month time point. A 5-point Likert Scale is used for each question, anchored at ‘1’ = strongly disagree, to ‘5’ = strongly agree;
vii) Self-efficacy for eating control assessed using the Weight Efficacy Lifestyle (WEL) questionnaire short form. Scored from 8 statements regarding eating control on a 10 point NRS [59] with total scores ranging from 0 to 80 and higher scores indicating greater self-efficacy for eating control;
viii) Arthritis self-efficacy scale short form [60] scored from 8 questions on a 10-point NRS with the total presented as an average of the 8 item scores and higher scores indicating higher self-efficacy;
ix) Physiotherapists’ confidence in their knowledge and in their clinical skills in weight management assessed using a custom-developed reliable and valid questionnaire [28] (knowledge score range 14–70 and skills score range 9–45, higher indicating greater confidence) before and after undergoing the training and after completion of the trial;
x) Physiotherapists self-rated confidence in delivering the ketogenic VLCD on an 11-point NRS anchored at 0 = no confidence to 10 = extremely confident at the end of the study.

### Baseline descriptive measures

Descriptive measures at baseline include: age; height; sex; education level; duration of symptoms; pain at other sites; current employment status; co-morbidities (evaluated using the Self-Administered Comorbidity Questionnaire [61]); dieting history (number of serious weight loss attempts over the last 5 years, reported in categories of 0, 1–2, 3–5, 5–10, more than 10); knee OA treatments used in past 6 months including pain medications; and expectation of treatment outcome (self-reported using a 5-point Likert scale with anchors of “no effect at all” to “complete recovery”).

### Co-interventions

Participants will self-report at 6 months any use of co-interventions (treatments) which they have used to manage their knee pain and reduce their body weight during the trial. Participants will also report at 6 months any pain and arthritis medications/supplements that they have taken at least once per week for their knee problem in the past month.

### Adverse events

Adverse events will be self-reported and are defined as any untoward medical occurrence in a participant that does not necessarily have a causal relationship with the treatment. Participants will be advised to report adverse events to the trial coordinator as soon as possible. Adverse events will also be self-reported in the 6-month questionnaire, where participants are asked to provide details of the nature of adverse event(s), how long they lasted for, what action, if any, they took (e.g. taking medication or seeing a healthcare professional), and whether they believe the adverse event was caused by participation in the trial (likely, unlikely, or unsure).

The chief investigator will determine whether the event is not related, related or possibly related to the participant’s involvement in the trial. Events considered to be related or possibly related will be recorded as related adverse events. Serious adverse events will be defined as any untoward medical occurrence that; i) results in death; ii) is life-threatening; iii) requires hospitalisation or prolongation of existing hospitalisation; iv) results in persistent or significant disability or incapacity; v) is a congenital anomaly or birth defect, or; vi) any other important medical condition which may require medical or surgical intervention to prevent one of the outcomes (i)-(v). We will report the number and proportion of participants who: withdraw from the trial due to a related adverse event; experience one or more serious related adverse events and their types; and experience one or more non-serious related adverse events and their types.

### Trial sample size

Clinical practice guidelines for knee OA recommend patients who have overweight or obesity should lose at least 5–7.5% of body weight [62]. We therefore powered the trial to detect a conservative between-group difference in weight loss of 5% of body weight assuming no change in weight in the exercise group based on previous research [13, 63]. While the between-participant standard deviation of percentage change in body weight was 5% in another study [63], we were more conservative and assumed a larger standard deviation of 7.5% given that our program has substantially less therapist contact (6 in ours compared with up to 12 individual sessions and 42 group sessions in the previous study [63]), which could result in more variation in response. For a power of 0.8 and a two-tailed significance level of 0.05, we required 37 participants per group. We increased this to 44 participants per group (88 in total) to allow for a 15% loss to follow up [34]. Since physiotherapists treat participants in both arms of the trial, we have not adjusted the sample size calculation for clustering by physiotherapist. Due to unknown correlations between percentage weight loss and baseline weight in this population, sample size calculations are conservative and do not account for the adjustment of baseline weight which will further increase the statistical power.

### Data analysis plan

A full statistical analysis plan will be made publicly available while blinded and prior to commencement of formal statistical analysis. A biostatistician (FM, under supervision of KEL) will analyze data blind to group name. Comparative analyses between groups will use intention-to-treat. Multiple imputation will be used to account for missing data if the proportion of missing data for the primary outcome is > 5%.

For the primary outcome, the difference in mean percentage change in body weight will be compared between groups using linear regression modelling adjusting for baseline weight and the stratification variables of sex and physiotherapist. Similar analyses will be conducted for continuous secondary outcomes. We will also calculate the proportion of participants achieving ≥5% and ≥ 10% loss of body weight in both groups. Binary outcomes will be compared between groups using log-binomial regression, adjusting for the stratifying variables of sex and physiotherapist, with results reported as risk ratios and risk differences. Counts and percentages of participants experiencing improvement will be reported in each intervention group. Should the log-binomial regression models fail to converge, logistic regression models adjusting for the stratifying variables will be fitted, with results reported as risk ratios and risk differences. In addition to the intention-to-treat effect, we will obtain the complier average causal effect by making use of the collected adherence data. For all between-group comparisons, 95% confidence intervals and *p*-values will be reported. Standard diagnostic plots will be used to verify model assumptions. It is not anticipated that any interim analyses will be performed in this trial.

### Nested qualitative evaluations

To inform implementation, approximately 15 participants from the diet plus exercise group and all physiotherapists will undergo semi-structured individual interviews to explore their experiences of and attitudes towards physiotherapists delivering a dietary weight loss program. Trial participants will be purposively sampled to provide a spread with regards to sex, age, extent of weight loss and reported satisfaction with the diet program. Questions will explore experiences, acceptability, barriers and facilitators to implementation/uptake and ease of integration into the clinical setting. Interviews will be audio-recorded, transcribed and analysed thematically. The qualitative methodology and findings will have its own ethics application and be reported separately from the main trial protocol and findings.

### Timelines

Ethical approval was obtained from the Human Research Ethics Committee of The University of Melbourne in October 2019. The trial was prospectively registered with Clinicaltrials.gov in Feb 2021. Physiotherapists were recruited and underwent training from March to July 2021. Participant recruitment commenced in October 2021. Covid-19 related restrictions meant that recruitment was delayed. Recruitment is expected to be completed by December 2022. The trial is due for completion by June 2023 when all participants will have completed 6-month data. Analysis of data is due for completion by end of Sept 2023.

### Dissemination

Trial findings will be disseminated through publication in peer-reviewed journals and conference presentations as well as via our Centre website, media and social media including a study infographic. No information which could lead to the identification of a participant will be included in the dissemination of results. Only fully non-identifiable data will be presented when disseminating results.

## Discussion

This protocol describes the background, aims and protocol for a two-group, parallel superiority RCT aiming to compare the addition of a ketogenic VLCD program to exercise delivered by physiotherapists via videoconference versus exercise alone, on weight loss in people with knee OA and overweight or obesity. The effects of the combined program on other clinical outcomes will also be evaluated. A range of process measures will provide insights into the participants’ experience with the diet program delivered by physiotherapists. The findings will help provide evidence as to the effectiveness, safety, feasibility and acceptability of this innovative model of service delivery for weight management that expands the scope of physiotherapists’ practice. Such a service model has the potential to increase patient access to evidence-based weight management programs.

## Data Availability

The deidentified datasets used and/or analysed during the current trial will be made available from the corresponding author on reasonable request.

## Abbreviations

AQoL: Assessment of Quality of Life
BMI: Body mass index
CONSORT: Consolidated Standards of Reporting Trials
CSIRO: The Commonwealth Scientific and Industrial Research Organization
GI: Glycemic index
GP: General practitioner
HREC: Human Research Ethics Committee
iCOAP: Intermittent and Constant Osteoarthritis Pain
NRS: Numeric Rating Scale
OA: Osteoarthritis
PASE: Physical Activity Scale for the Elderly
RACGP: The Royal Australian College of General Practitioners
RCT: Randomized Controlled Trial
SPIRIT: Standard Protocol Items: Recommendations for Interventional Trials
TIDieR: Template for Interventional Description and Replication
VLCD: Very Low Calorie Diet
WEL: Weight Efficacy Lifestyle
WOMAC: Western Ontario and McMaster Universities Osteoarthritis Index
WSSQ: Weight Self-Stigma Questionnaire
